# Characterization of Cure Behavior in Epoxy Using Molecular Dynamics Simulation Compared with Dielectric Analysis and DSC

**DOI:** 10.3390/polym13183085

**Published:** 2021-09-13

**Authors:** Shuang Yan, Wolfgang Verestek, Harald Zeizinger, Siegfried Schmauder

**Affiliations:** Institute for Materials Testing, Materials Science and Strength of Materials, University of Stuttgart, Pfaffenwaldring 32, 70569 Stuttgart, Germany; wolfgang.verestek@imwf.uni-stuttgart.de (W.V.); zeizinger@arcor.de (H.Z.); siegfried.schmauder@imwf.uni-stuttgart.de (S.S.)

**Keywords:** epoxy, curing, reaction kinetics, molecular dynamics, dielectric analysis (DEA), differential scanning calorimetry (DSC)

## Abstract

The curing behavior of a thermosetting material that influences the properties of the material is a key issue for predicting the changes in material properties during processing. An empirical equation can describe the reaction kinetics of the curing behavior of an investigated material, which is usually estimated using experimental methods. In this study, the curing process of an epoxy resin, the polymer matrix in an epoxy molding compound, is computed concerning thermal influence using molecular dynamics. Furthermore, the accelerated reaction kinetics, which are influenced by an increased reaction cutoff distance, are investigated. As a result, the simulated crosslink density with various cutoff distances increases to plateau at a crosslink density of approx. 90% for the investigated temperatures during curing time. The reaction kinetics are derived according to the numerical results and compared with the results using experimental methods (dielectric analysis and differential scanning calorimetry), whereby the comparison shows a good agreement between experiment and simulation.

## 1. Introduction

Thermoset materials are widely used in the industrial sector because of their excellent mechanical properties at high temperatures and good chemical resistance. The non-cured thermosetting resin contains many monomers, which crosslink from a specific temperature and build three-dimensional macromolecules during processing. Since it causes changes in dynamical viscosity and temperature in the material, it is essential to know how the cure behavior changes during processing, e.g., the injection molding process. Several experimental methods are available for monitoring and investigating the thermoset material’s cure behavior: differential scanning calorimetry (DSC), dielectric analysis (DEA), Fourier transform infrared spectroscopy (FTIR), near-infrared spectroscopy (NIRs), and ultra-sonic techniques. However, using the measurement technology to monitor curing involves a high cost and effort and, in terms of the manufacturing process, the sensors cannot be adopted at every position where the curing state in the material needs to be measured. The molecular changes in morphological structure caused by the curing, which influence the material properties, are more easily predicted and analyzed with the help of molecular dynamics (MD) simulation.

Molecular dynamics simulation is commonly used in thermoset polymers for material design and to predict material properties. The previous studies [1,2,3,4,5,6,7,8,9,10,11,12,13,14,15] focused on predicting the thermomechanical properties (e.g., glass transition temperature, Young’s modulus, thermal conductivity, and compressibility) in already cured molecular structures of the epoxy polymers using *MD* simulation. The molecular structures (Li et al. [4]) and the crosslink density (Schichtel et al. [10]) strongly influence the thermomechanical properties. Okabe et al. [9] investigated the curing behavior of diglycidyl ether bisphenol A (*DGEBA*), tetra glycidyl diamino diphenyl methane (*TGDDM*), and eight polymers of diglycidyl ether bisphenol A (*DGEBA8*) as base resins, with 4′4-diaminodiphenylsulphone (*44DDS*) and 3′3-diaminodiphenylsulphone (*33DDS*) used as curing agents. The simulations showed an acceptable comparability of curing curves in different resin mixtures computed using *MD* simulation with experimental *DSC* measurements. Although the curing behavior was simulated in this study, it focused more on the resulting mechanical properties caused by different functional groups. In the study from Li et al. [16], the model was extended to a hybrid molecular dynamics/Monte Carlo (*MD*/*MC*) approach with reaction probabilities based on reaction path energies. Reactions of phenol resin with formaldehyde are investigated, and several thermal-mechanical properties agree with the experimental values.

Unger et al. [17] attempted to validate the molecular modeling of a pure epoxy resin (base resins: bisphenol A diglycidyl ether, 1,4-Butanediol diglycidyl ether, Alkyl (C12-C14) glycidyl ether; hardener: α,ω-poly(oxypropylene)diamine, 3-(aminomethyl)-3,5,5-trimethyl-cyclohexanamine, 2,4,6-tris[(dimethylamino)methyl]phenol) according to the spectroscopic changes in correlation with crosslink density by means of in situ *NIR*, which showed a good agreement between numerical and experimental results.

The above-mentioned studies have shown that *MD* can simulate the curing of epoxy networks. However, the cure behavior (reaction kinetics) for different material systems was not investigated in detail because the curing was considered simply as a modeling step, and only the thermomechanical properties were checked. Either that or united atom force fields, e.g., the *DREIDING* force field, were used, which has been shown by Li et al. [18] to result in different morphologies than all-atom force fields.

Until now, a validation of molecular modeling for curing using experimental methods is still challenging due to the difference in time scale between the molecular dynamics simulation in the nanoseconds range and the experimental study in the seconds range. This work focuses on analyzing the cure behavior of an epoxy resin (base resin: bisphenol A diglycidyl ether (*BADGE*) and hardener: ethylenediamine (*EN*)) with consideration of the thermal influence of using *MD* simulation. In addition, the accelerated reaction kinetics influenced by an increased reaction cutoff distance are investigated. Finally, the numerical results are compared with experimental results derived by *DSC* and *DEA,* whereby *DSC* is only applicable in a laboratory environment and *DEA* is suitable for in situ cure monitoring. A commercial epoxy composite applied in industrial application is measured in our experimental investigations, whose polymer matrix (base resin and hardener) is modeled in the *MD* simulation. Reaction models as part of reaction kinetics for describing the cure mechanism are estimated simultaneously using *MD*, *DSC* and *DEA* methods for evaluating their comparability.

## 2. Materials and Methods

### 2.1. Material

The resin system, also referred to as *EP* in the following text, consists of epoxy resin bisphenol A diglycidyl ether (*BADGE*) and a hardener, ethylenediamine (*EN*), that crosslink via a polymerization reaction (see Table 1). In this molecular modeling, three possibilities for the crosslinking reaction baetween the functional groups are considered:

(a) Reaction between the epoxide group (*R*_1_*-CCO*) and the primary amine group (*R*_2_*-NH*_2_);
(b) Reaction between the epoxide group (*R*_1_*-CCO*) and the secondary amine group (*R*_3_*-NH-R*_4_);
(c) Reaction between the epoxide group (*R*_1_*-CCO*) and the hydroxyl group (*R*_5_*-OH*).

**Figure A1:**
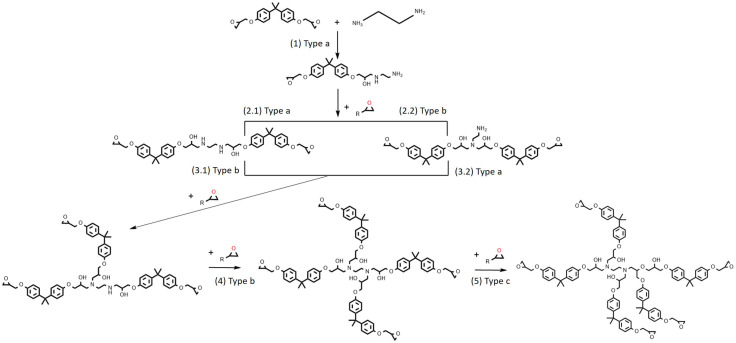
Schematic representation of the crosslinking reaction between (a: epoxide group and primary amine group, b: epoxide group and secondary amine group, and c: epoxide group and the hydroxyl group).

The detailed sequence of the crosslinking reaction with molecular structures as examples, is shown in Figure A1 in Appendix A.

### 2.2. Molecular Modeling

For computing the chemical reactions (bond breaking, bond formation, and reactivity) and predicting the material properties, there are two types of force fields: fixed force fields (also called classical force fields) and reactive force fields. For classical force fields (e.g., *CVFF*, *PCFF*, *DREIDING*, *COMPASS*), bonds are defined explicitly, while maintaining a low computing time and good accuracy [1,3,11,12,19,20,21,22]. In studies [8,23], a reactive force field (*ReaxFF*) is used to simulate the chemical reaction. The advantage of *ReaxFF* is that the bond formation is defined implicitly and happens dynamically during computing, which needs a very high computing capacity and a high complexity of parameterization. In this study, the crosslinking reactions between the functional groups are implemented in molecular modeling using a fixed force field (*PCFF*).

The software *EMC* (Enhanced Monte Carlo), developed by Pieter J. in ’t Veld [24], and *LAMMPS* Simulator (Large-scale Atomic/Molecular Massively Parallel, Sandia National Laboratories, USA) [25,26,27] were applied to prepare the initial molecular structures and molecular modeling.

- Step 1. Generation of the initial molecular network of the uncured resin system.

The first step was to generate the initial stochastic mixture of the uncured resin system using *EMC* software. *EMC* followed a combined Monte Carlo/MD approach. The fixed force field *PCFF* (Sun et al. [28]) was used to parameterize intra- and inter-molecular interactions between the atoms. The ratio of epoxy resin *BADGE* to the hardener *EN* was 2:1. The epoxy resin *BADGE* amount is *N_BADGE_* = 1817, which included the amount of the epoxide group *N_-CCO_* = 3634. The amount of the hardener *EN N_EN_* = 908, and includes the primary amine group *N_-NH2_* = 1818. The initial density after creation by *EMC* was 1 g/cm^3^.

As an initial equilibration, a short simulation sequence in microcanonical ensemble *NVE* (with limited maximum displacement per step), canonical ensemble *NVT,* and isothermal-isobaric ensemble *NPT* was performed in *LAMMPS* with a time step of 0.25 fs, at a target temperature of 150 °C, and for a total time of 25 ps.

- Step 2. Thermodynamical equilibration of the initial uncured molecular network.

The initial uncured molecular mixture was equilibrated for 150ps under periodic boundary conditions using *NPT* to achieve thermodynamical equilibrium. A time step size of 1.5 fs was applied for these and all following simulations by utilizing the rRESPA algorithm (Plimpton et al. [26]) implemented in *LAMMPS*. The conditions (pressure *P*: 423 atm; temperature *T*: 150 °C, 170 °C, and 190 °C), which were applied as process parameters in *DEA* measurements in an actual injection mold, were implemented for the equilibration procedure. Table 2 lists the density of the uncured molecular system in a simulation box (including its dimension and volume) after equilibration at three temperatures.

- Step 3. Identification of the reactive bonding and definition of criteria for the beginning of crosslinking.

For crosslinking resin and hardener, the REACTER protocol [29,30] as implemented in *LAMMPS* was used. The cutoff distance *D_cut_*, which correlated to the bond energy, was defined as a criterion for bond breaking and creation. Bond dissociation energy described the amount of energy that was required to split the bond homolytically. Numerically, the reactivity of the crosslinking reaction was changed through varying the cutoff distance *D_cut_*. By increasing cutoff distance, the reactivity increased, and the duration until the material was fully crosslinked reduced. For example, during curing between epoxide and amine groups, if the distance between functional groups satisfied the boundary condition (≤cutoff distance *D_cut_*), the bond *C-O* in the epoxide group will break up and link up to the bond *N-H*, which also breaks simultaneously.

As a reference, the bond dissociation length of the newly created bonds (*O-H*, *C-N*, and *C-O*) was calculated using the class2 force field (see Table 3). The bond potentials *V_bond_* between the atom *i* and *j* were generated with Equation (1) using class2 bond style presented by Sun [31]:

(1) $$V_{bond,ij}\left(r_{ij}\right)=K_{2}\cdot {\left(r_{ij}-r_{ij.0}\right)}^{2}+K_{3}\cdot {\left(r_{ij}-r_{ij.0}\right)}^{3}+K_{4}\cdot {\left(r_{ij}-r_{ij.0}\right)}^{4}$$

Parameter $r_{ij.0}$ presents the equilibrium bond distance. *K*_2_, *K*_3_, and *K*_4_ are bond coefficients.

This molecular modeling of crosslinking performed using the classic force field showed several advantages. It needed less computing capacity and performed more efficiently. The temperature influence and the effect of cutoff distance *D_cut_* on the cure kinetics were analyzed. The curing process was simulated at temperatures of 150 °C, 170 °C, and 190 °C while varying the cutoff distance *D_cut_* of the *O-H* bond from 1.5 Å→2.5 Å.

### 2.3. Dielectric Analysis

The dielectric analysis is a measuring procedure to study the material’s response to an applied electric field. The electrical current *I_electr_(f)* flowing through an electric capacitor, in which the epoxy material as dielectric material was located, was measured as a response to an alternating electric field depending on field frequency *f*. This analyzed the changes in dielectric properties, e.g., the relative permittivity *ε_r_* of the polymer, depending on the changes in molecular and morphological structures in the material. Therefore, the the dialectric behavior of thermosets can be used to monitor the curing process which causes changes in dielectric properties. Permittivity *ε*, determined by the material polarization in response to the electrical field, is a physical quantity describing the interaction between the electric field and the dielectric material. Relative permittivity *ε_r_* is defined as the ratio of dielectric permittivity *ε* to vacuum permittivity *ε*_0_.

The electric field polarized the dipoles and freed the charges in the investigated material, leading to a change in relative permittivity related to the curing. The relative permittivity was calculated according to the measured electrical current and electrical voltage. The electrical current *I_electr_* resulted from dipole interaction in the capacitor’s electric field and the mobility of ionized molecules. The relative permittivity *ε_r_* of the investigated material was a complex quantity with angular frequency *ω = 2πf*, shown in Equation (2).

(2) $$\varepsilon_{r}\left(\omega \right)=\varepsilon^{\prime }\left(\omega \right)-i\cdot \varepsilon^{\prime \prime }\left(\omega \right)$$

The term *ε’* is the real part of relative permittivity that represents stored electric energy in the material. The imaginary part *ε”* of the relative permittivity (dielectric loss) describes the lost energy of the applied external electric field.

**Figure 1:**
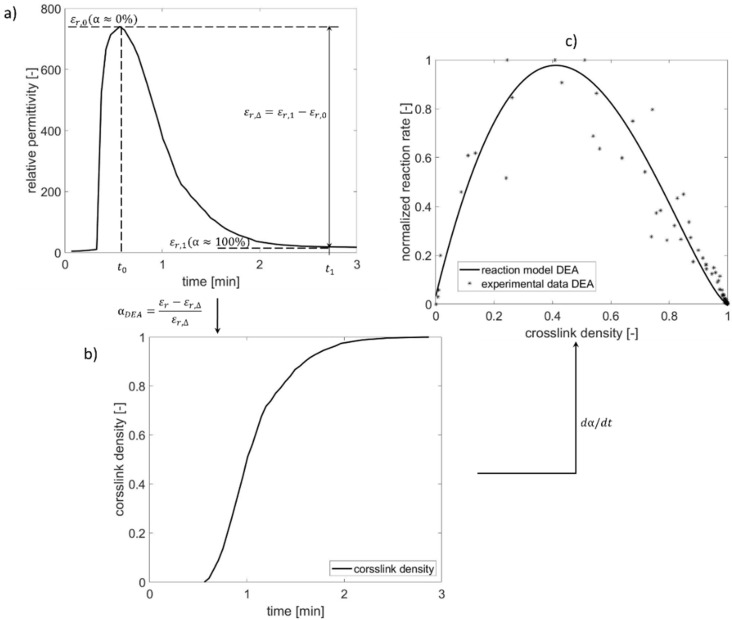
Evaluation procedure of *DEA* result: (**a**) maximum and minimum of relative permittivity, (**b**) crosslink density based on *DEA* result, and (**c**) reaction rate in dependency of crosslink density.

In this study, dielectric measurements were performed using a sensor TMC 16/3 (NETZSCH-Gerätebau GmbH, Selb, Germany) with 1 kHz at a pressure of 423 atm and temperatures of 150 °C, 170 °C, and 190 °C. Figure 1 shows an example of the evaluation procedure of a *DEA* measuring result obtained at the temperature of 150 °C. The change of relative permittivity *ε_r_* during heating is shown in Figure 1a. Firstly the relative permittivity *ε_r_* increases until *t*_0_, which indicates the melting of the materials and the increased amount of available charge carriers in the material. After the onset of curing, the polymer system transits from a highly viscous to solid-state, and the content of free movable charges reduces, leading to a decrease in the relative permittivity *ε_r_* and an increase in crosslink density, meaning that *ε_r_* ~ *1/α*. The maximal relative permittivity *ε_r,0_* at *t*_0_ means the curing begins (*α* ≈ 0%). After the material is almost fully cured, the relative permittivity *ε_r_* reaches a plateau. Termination of curing (*α ≈* 100%) is determined at time *t_1_* when the relative permittivity’s change rate is less than 1%. Thereby, the value of relative permittivity *ε_r,_*_1_ is determined at time *t*_1_. Based on the relative permittivity’s change in correlation with crosslink density *α* during curing, the crosslink density is estimated as a function of heating time according to Equation (3), shown in Figure 1b. In the further step, the reaction rate *dα/dt* in dependency on the crosslink density *α*, shown as dots in Figure 1c, can be derived regarding the results of the last step to determine the unknown parameter in reaction model *f(α)*. Whereby the black curve of *f(α)* is the fitted line regarding the values of *dα/dt* vs. *α*.

(3) $$\begin{array}{c} \alpha_{DEA}=\left(\varepsilon_{r}-\varepsilon_{r,∆}\right)/\varepsilon_{r,∆}\\ \varepsilon_{r,∆}=\varepsilon_{r,1}-\varepsilon_{r,0} \end{array}$$

*α_DEA_* is the crosslink density determined using *DEA*. The term *ε_r,_*_0_ means the maximal relative permittivity occurring at *t*_0_; *ε_r,_*_1_ is the relative permittivity determined at time *t*_1_, when the reaction is terminated, and *ε_r,∆_* is the value of the difference between *ε_r,_*_0_ and *ε_r,_*_1_.

### 2.4. Differential Scanning Calorimetry

*DSC* is a classic method for analyzing the correlation between the curing amount, temperature, and time, based on the amount of heat released from crosslinking reactions. The equipment DSC Q100 (TA Instruments–Waters LLC, New Castle, DE, USA) was used for studying the reaction kinetics. Non-isothermal measurements were performed with heating rates of 2 °C/min, 5 °C/min, 10 °C/min, 15 °C/min, and 20 °C/min with nitrogen *N*_2_ as the purge gas.

**Figure 2:**
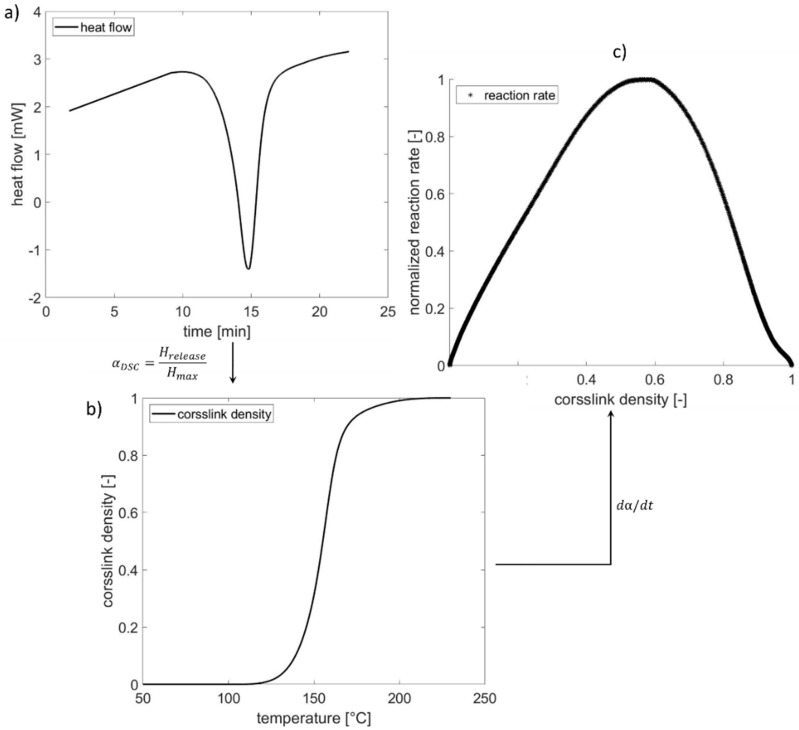
Evaluation procedure of *DSC* result measured under the non-isothermal condition: (**a**) change of heat flow during the curing process with heating rate 10 °C/min, (**b**) crosslink density, and (**c**) reaction rate in dependency of curing amount.

The thermally stimulated depolarization currents method [33,34] and *DSC* are applicable for an experimental determination of the activation energy, while the *DSC* method has been employed in our study. Concerning the results of *DSC* measurements, the activation energy *E_A_* and the pre-exponential *A* could be determined using the Kissinger method [35]. For predicting the unknown parameter in reaction model *f(α*), the following procedure is performed. During the curing process, the change of heat flow in dependency of time and temperature is detected by *DSC* shown in Figure 2a. According to the resultant change of heat amount, temperature- and time-dependent crosslink density is a ratio of the released heat amount *H_release_* to the maximum heat amount *H_max_* in a fully crosslinked state, using Equation (4) (see Figure 2b). Figure 2c shows the reaction rate in dependency on the crosslinking degree to estimate the unknown parameter in the reaction model *f(α)*:

(4) $$\alpha_{DSC}=H_{release}/H_{max}$$

The term *α_DSC_* represents the crosslink density determined using *DSC*. The *H_release_* is the released heat amount by the material in the partially cured state, and *H_max_* is the maximum heat amount released by the material in the fully cured state.

## 3. Results and Discussion

### 3.1. Curing Behavior

**Figure 3:**
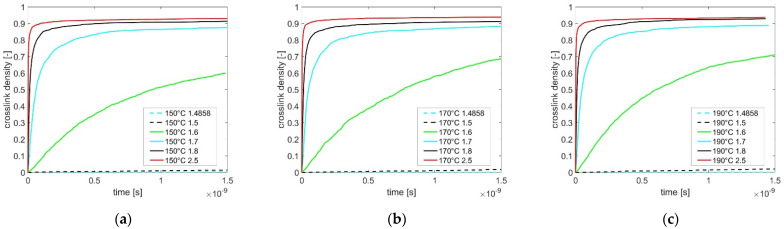
Curves of crosslink density depending on curing time concerning the influences of cutoff distances (1.48 Å→2.5 Å) at (**a**) 150 °C, (**b**) 170 °C, and (**c**) 190 °C.

Once the initial uncured molecular mixtures were finished to equilibrate with *NPT*, the crosslinking simulations were performed with varied cutoff distances for *O-H* bonds from 1.48 Å up to 2.5 Å at 150 °C, 170 °C, and 190 °C (shown in Figure 3a–c). The number of completed reactions is counted during simulation. Since the epoxide group *-CCO* participates in each of the modeled crosslinking reactions, Equation (5) is derived for estimating the crosslink density as the ratio of the number of already utilized epoxide groups *N_u,CCO_* to the total number of epoxide groups *N_total,CCO_*.

The computing time and effort, with a calculated bond dissociation length (*D_cut_* = 1.48 Å), are enormous until the material is fully cured as obtained by the *MD* simulation. Then, further simulations are carried out with the increasing cutoff distance *D_cut_* of 1.48 Å up to 2.5 Å, which increases the reactivity of the chemical reactions, respectively.

Based on computing results, the cutoff distance has a significant influence on the curing reaction’s activity. At three temperatures, the molecules crosslink very slowly with a low cutoff distance of 1.5 Å, and it is to be expected that a significant change in crosslink density is only visible over a very long simulation time. However, since the cutoff distance is set to larger than 1.6 Å, the crosslinking process becomes significantly faster. Additionally, the maximal achieved crosslinking amount also increases. A similar effect of cutoff distance on the crosslinking behavior is also observed in the report by Unger et al. [17].

Generally, a higher environment temperature (e.g., 190 °C) excites stronger molecular oscillations, which cause a higher molecular diffusion in the simulation system. Thus, while the molecular movement becomes more active at a higher temperature of 190 °C than at a lower temperature of 150 °C, the epoxy resin, over time, has a higher probability for meeting a hardener and linking up. Respectively, an achievement of a higher crosslink density at a higher temperature is expected, which is demonstrated in our simulation study with the cutoff distance of 1.6 Å. However, the influence of the rising environmental temperature on the crosslink density is no more significantly recognized in respect of numerical results computed with a cutoff distance of ≥ 1.7 Å.

As described in step 2 in Section 2.2, the molecular mixture is previously equilibrated at the given temperature, and all molecules distribute homogeneously in space. Since the diffusion of molecules is a long-term process which only occurs at a lower cutoff distance (e.g., 1.6 Å), indicating a longer curing time until the maximum crosslink density is achieved, the influence of the increasing temperature (150 °C→190 °C) on diffusion and the corresponding crosslink density is observed in Figure 3. By increasing the cutoff distance (≥ 1.7 Å), the molecules have a higher probability of reacting with the neighboring reaction partners in a homogenized polymer mixture, while the simulated curing time is reduced, therefore preventing molecular diffusion. As a result, excited intermolecular interactions are not significantly observable due to the increasing temperature, which leads to a similar crosslink density value simulated with the equal cutoff distance at varying temperatures (150 °C→190 °C).

Additionally, the molecular simulation demonstrates that a fully cured molecular network (*α* ≈ 100%) is hardly realizable. During the curing process, more crosslinked molecular networks are created and grow continuously to a larger size. The large molecules restrict the remaining epoxy resin and hardener from free movement and finding the reaction partner.

(5) $$\alpha_{MD}=\frac{N_{u,CCO}}{N_{total.CCO}}$$

The term *α_MD_* represents the crosslink density determined using molecular dynamics simulation. *N_u,CCO_* is the number of epoxide groups already utilized during curing, and *N_total,CCO_* is the total number of epoxide groups.

Table 4 lists the densities of the molecular mixtures at the end of the curing simulations for cutoff distances (1.48 Å→2.5 Å) at 150 °C, 170 °C, and 190 °C. Referenced to the initial densities of the uncured molecular system in Table 2, the molecular mixture’s density at the end of simulation increases when the achieved crosslink density grows by increasing the cutoff distances shown in Figure 3. Additionally, the density decreases during the increasing temperature of 150 °C→190 °C, which correlates to the thermal expansion of the molecular system influenced by a higher temperature.

**Figure 4:**
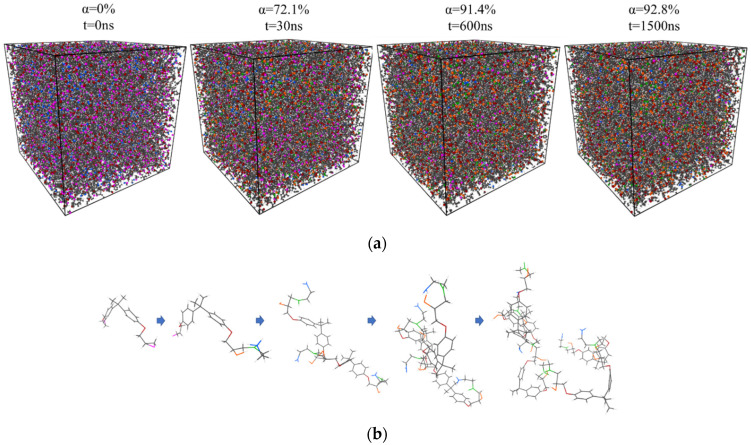
Changes in molecular structures during the curing process with the cutoff distance of 1.8 Å at 190 °C: (**a**) total view of the simulation box with periodic boundary, and (**b**) growth of the molecule as an example.

Changes in the molecular structures regarding the result with a cutoff distance of 1.8 Å at 190 °C are schematically shown as an example in Figure 4. The colors of electrically charged atoms change according to changes in the atoms’ charges during bond breaking and formation.

**Figure 5:**
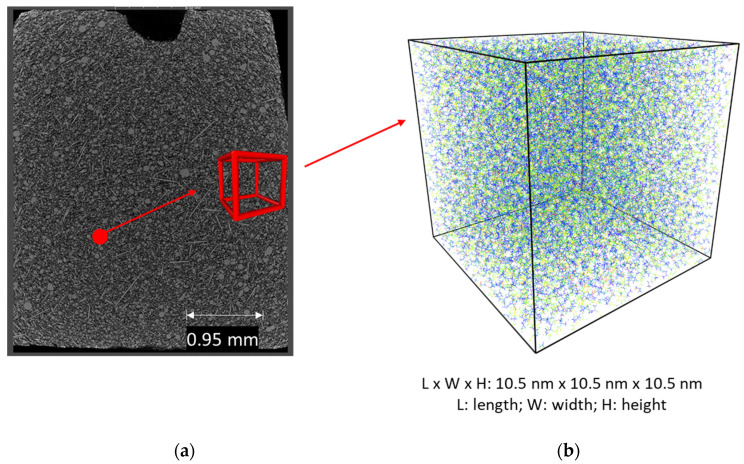
Macro- and micro-structure of the epoxy resin: (**a**) Nano-CT of the epoxy resin, and (**b**) view of simulation cell in molecular dynamics simulation.

The reaction speed simulated by molecular dynamics is significantly higher than the experimentally determined value. In contrast to the experiment in which a larger test specimen is considered in millimeter scale, the material volume in nanometer-scale is taken into account in *MD* simulation, shown in Figure 5. An ideal thermodynamic state is set in molecular dynamics, under a constant pressure and temperature with a periodic boundary condition, meaning that there is no heat transfer within the material and no heat loss occurs between the edge and the environment. In the experimental analysis, heat transfer within the material and thermal exchange within the environment causes the imbalance of the temperature distribution across the layer thickness in the polymer matrix from *EP*. Additionally, monomers are homogeneously distributed in the simulation volume, leading to a shorter balancing time of the molecular movements and a faster reaction. Furthermore, besides pure epoxy resin and hardener, the commercial *EP* material investigated in the experiment contains additives such as the reaction inhibitor, which delays and slows down the curing reaction.

### 3.2. Reaction Kinetics Using Molecular Dynamics Simulation

Reaction kinetics describe the rate of the chemical reaction, which considers temperature, pressure and concentration dependency: *k(T)*, *f(α),* and *h(p)* (see Equation (6)).

(6) dα/dt=k(T)·f(α)·h(p)

Supposing that the pressure *p* is kept constant during the chemical reaction process, the pressure dependency can be ignored, and Equation (6) is simplified into Equations (7) and (8).

(7) dα/dt=k(T)·f(α)

The temperature dependency, *k(T) = A·exp(-E_A_/(R_gas_·T))*, generally known as the Arrhenius equation, consists of the material-related activation energy *E_A_* and the pre-exponential term *A*:

(8) $$d\alpha /dt=A\cdot exp\left(-E_{A}/\left(R_{gas}\cdot T\right)\right)\cdot f\left(\alpha \right).$$

The reaction model *f(α)* describes the reaction mechanisms in dependency on the proportion of crosslinked molecules. There are already empirical equations available (such as *nth*-order catalytic, Avrami–Erofeev, Prout–Tompkins, and Sestak–Berggren reaction model [36,37]), whose unknown factors can be determined by regression methods. Since the empirical model (*nth*-order catalytic) best fits the numerical results for this investigated material, only this model is considered further in our study.

**Figure 6:**
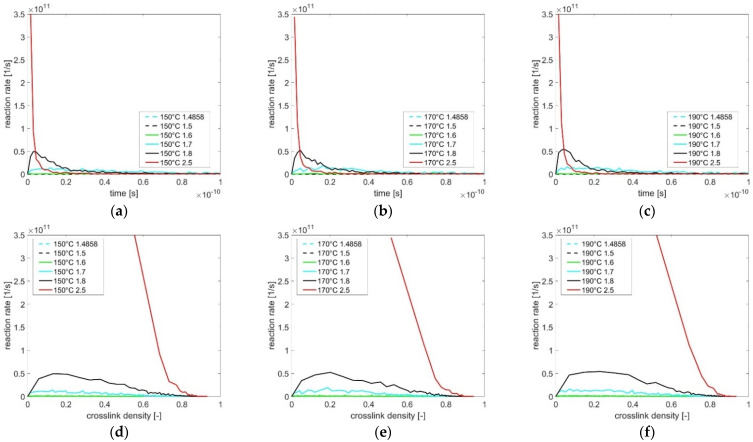
(**a**–**c**) Curves of reaction rate depending on curing time and (**d**–**f**) curves of reaction rate depending on crosslink density concerning the influence of cutoff distance (1.48 Å→2.5 Å) at 150 °C, 170 °C, and 190 °C.

**Figure 7:**
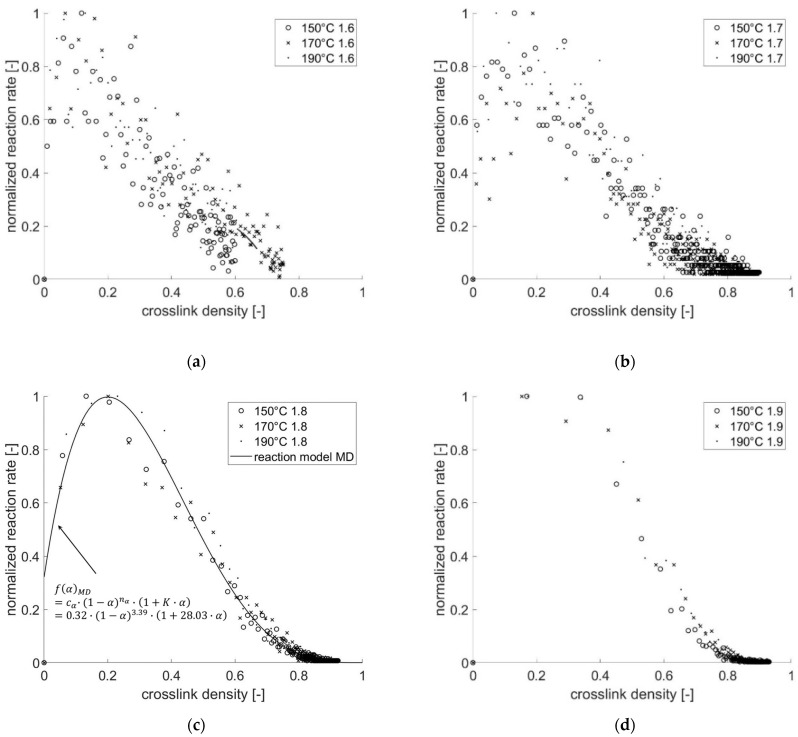
Reaction rate depending on crosslink density computed using varied cutoff distances of (**a**) 1.6 Å, (**b**) 1.7 Å, (**c**) 1.8 Å and (**d**) 1.9 Å.

Concerning the difference in the time scale between the molecular dynamics simulation in the nanoseconds range and the experimental study in the seconds range, the evaluation procedure for determining the reaction model *f(α)* regarding the experimental results can also be applied for the calculation based on the simulation results. As shown in Figure 6a–c, the time dependent reaction rate is calculated based on crosslink density depending on curing time, which is simulated using various cutoff distances and temperatures in Figure 3. With a large cutoff (2.5 Å), the maximal reaction rate is achieved immediately after the beginning of curing. As the cutoff distance decreases, the maximal reaction rate decreases until no reaction occurs during the simulation time. The data of reaction rate and the associated crosslink density at the same curing time are transferred to Figure 6d–f to further explain the determination of the unknown parameters in reaction model *f(α)*. After that, the reaction rate curve is normalized according to the maximum reaction rate, related to the associated cutoff distance and temperature. Since the temperature dependency is considered by factor *k(T)* in Equation (7), the data (normalized *dα/dt* vs. *α*), simulated with the same cutoff distance at different temperatures, are merged for the subsequent regression procedure (see Figure 7).

Figure 7 shows the normalized reaction rate in the dependency of crosslink density computed using different cutoff distances. The plot of reaction rate scatters less by increasing cutoff distance. However, supposing that the cutoff distance is set too high, such as at 1.9 Å, the chemical reaction takes place too fast so that the maximum reaction rate is already reached, even at a low crosslink density (Figure 7d). The reaction model *f(α)* is estimated based on the numerical results (normalized *dα/dt* vs. *α*) with the cutoff distance of 1.8 Å, whereby the regression result of the reaction model (*nth*-order catalytic) is presented in Figure 7c.

### 3.3. Comparison between Simulation and Experimental Results

#### 3.3.1. Reaction Kinetics Using *DEA*

**Figure 8:**
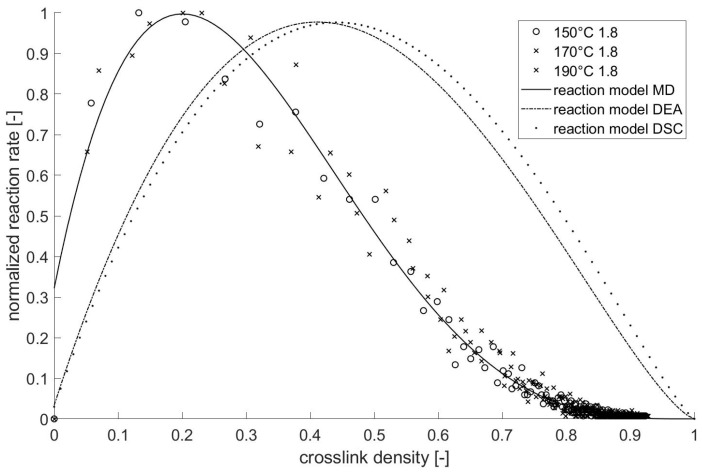
Comparison of the kinetic model derived using *MD*, *DEA*, and *DSC* methods.

*DEA* measurements were performed under the pressure of 423 atm and at temperatures of 150 °C, 170 °C, and 190 °C. The change in reaction rate depending on the crosslinking amount, which is calculated according to the relative permittivity’s changes at different temperatures, is plotted in Figure 8. At the beginning of curing (*α* ≈ 0%), a large number of ionized charge carriers in an alternating electrical field lead to the maximal relative permittivity in the material. The number of ions reduces, and the ions’ mobility is restricted strongly by the increasing crosslink density, reflecting the reduced relative permittivity in a *DEA* measurement. The relative permittivity converges when the material almost finishes curing (*α* ≈ 100%), even though the small content of reaction agents that cannot find a reaction partner is still available. The regression results using Trust-Region algorithms for determining the unknown parameters regarding the *DEA* measurements are listed in Table 5.

#### 3.3.2. Reaction Kinetics Using *DSC*

Concerning *DSC* measurements, the unknown parameters in Equation (8) are determined in two steps. First, the reaction model *f(α)* is fitted with the Trust-Region algorithm, whereby the results are listed in Table 5. Figure 8 shows the fitted curve regarding the *nth*-order catalytic reaction model to the reaction rate, resulting in the dependency of the crosslinking amount measured with different heating rates *β*. Second, the Kissinger method was applied for determining the activation energy *E_A_* and the pre-exponential term *A*. Equation (9) can be evaluated according to a linear relationship $y=a_{K}\cdot x+b_{K}$, reported by Yan et al. [38]. Then, the activation energy *E_A_* and the pre-exponential term *A* are determined using Equations (10) and (11):

(9) $$ln\left(\frac{\beta }{T_{P}{}^{2}}\right)=-\frac{E_{A}}{R_{gas}\cdot T_{P}}+ln\left(-\frac{A\cdot R_{gas}}{E_{A}}\cdot f^{\prime }\left(\alpha_{P}\right)\right)$$

(10) $$a_{K}=-\frac{E_{A}}{R_{gas}}=>\;E_{A}=77.66\;kJ/mol$$

(11) $$b_{K}=ln\left(-\frac{A\cdot R_{gas}}{E_{A}}\cdot f\prime \left(\alpha_{P}\right)\right)=\text{> }A=\frac{-10^{b_{K}}\cdot E_{A}}{R_{gas\cdot f\prime \left(\alpha_{P}\right)}}\;1/s$$

where $f^{\prime }\left(\alpha \right)=df\left(\alpha \right)/d\alpha$, *R_gas_* is the gas constant, *β* is the heating rate, *T_p_* represents the temperature, and *α_p_* is the crosslink density with the index *p*, which means that the indicated values correspond to the position of the rate peak maximum.

#### 3.3.3. Comparison

The results of the reaction model determined by different methods (*MD*, *DSC*, and *DEA*) are transmitted together in the Figure 8 (normalized *dα/dt* vs. *α*) and Table 5. While the maximum response rate appears at a low network level (approx. 20%) in the *MD* simulation, the maximum reaction rate occurs at a crosslink density of 50%, concerning experimental results. According to the *MD* calculation, the crosslinking reaction ends almost at a high crosslink density of >85%. The mobility of free monomers/molecules is high at a low crosslink density, leading to the increased probability of the crosslinking reaction occurring. The barrier that prevents the molecular movements through morphological structures by the growing molecules is proportional to the increasing crosslink density. In an experimental investigation like *DSC*, the reaction heat is measured to determine the crosslink density. The maximal crosslink density determined using experimental methods is 100% since it assumes that all monomers/functional groups meet a reaction partner. Additionally, the phase transition from solid to liquid during the melting phase is not considered to be using molecular dynamics. Therefore, after the phase change in the material, the movement of the reaction partners increases in the fluid state. Furthermore, the mixture of pure resin and hardener is modeled in molecular dynamics, without considering the additives’ molecules which are commonly applied in a polymer blend for influencing the reaction speed or material properties. However, the comparison shows a good comparability and match between simulation and experimental results.

## 4. Conclusions

In this work, molecular dynamics simulation was applied to depict the hardening process of an epoxy resin at the atomic level using a fixed force field *PCFF* under periodic boundary conditions with constant pressure and at different temperatures. The reactivity of the functional groups is varied through changing cutoff distance. In the first step, the influence of cutoff distance (1.48 Å→2.5 Å) and temperature (150 °C→190 °C) on the material’s curing is investigated. The numerical results show that the maximal achieved crosslink density increases with the growing cutoff distance during simulation time. The crosslink density increases up to a maximum value (e.g., approx. 90% at a cutoff distance of 1.8 Å) and then remains almost constant, demonstrating that a fully crosslinked state can hardly be reached. Additionally, the enhanced molecules’ diffusions due to increasing the temperature from 150 °C to 190 °C is significantly recognized in the curing computed with a low cutoff distance (e.g., 1.6 Å). However, the influence of rising environment temperature on the crosslink density is not significantly identified regarding numerical results computed with a cutoff distance of ≥ 1.7 Å. This is because the molecules have a higher probability of reacting with the neighboring reaction partners in a homogenized polymer mixture and the curing time is too short for the diffusion process, respectively. In the further step, the reaction model *f(α)*, as a part of reaction kinetics, was determined based on the numerical results of the molecular dynamics study and compared to the experimental results determined by the *DSC* and *DEA* method. This approach indicates that the difference in the time scale between molecular dynamics and experiment need not be considered by adjusting the reaction cutoff distance and accepting accelerated reaction dynamics. The comparison demonstrates a similar course of the reaction model between simulation and experiment, while the maximum reaction rate in the *MD* simulation shifts to a lower crosslink density than the experiment. Nevertheless, the results show the feasibility of using molecular dynamics to derive reaction kinetics for a simple polymer system.

In future investigations, the influences of chosen cutoff distances on the topology of the generated epoxy networks, and therefore the associated material’s mechanical properties, should be analyzed in detail. Aside from predicting the material properties, molecular dynamics simulation offers a possibility for estimating the maximum achievable crosslink density, which is expensive to measure in experiments. In addition, the determined reaction model combined with the activation energy can be implemented in a macroscale process simulation, e.g., using the *FE* method.

## Figures and Tables

**Table 1:** Overview of the resin system.

	Epoxy Resin	Hardener
Chemical formula	bisphenol A diglycidyl ether (*BADGE*)	ethylenediamine (*EN*)
Molar mass	340.419 g/mol	60.1 g/mol
Molecular formula	C_21_H_24_O_4_	C_2_H_8_N_2_
Chemical structure	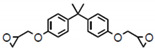	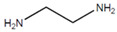

**Table 2:** The density of the initial molecular mixture in a simulation cell equilibrated at 150 °C, 170 °C, and 190 °C.

T [°C]	Initial Density [g/cm^3^]	Initial Dimension [nm^3^]	Initial Volume [nm^3^]
150	0.988	10.4 × 10.4 × 10.4	1131.3
170	0.977	10.4 × 10.4 × 10.4	1144.2
190	0.962	10.5 × 10.5 × 10.5	1162.1

**Table 3:** The bond dissociation length of the newly created bonds.

Bond-Type (*i*– *j*)	Unit	O–H	C–N	C–O
Bond dissociation energy $V_{bond,ij}$ [32]	[kJ/mol]	463	305	358
Bond dissociation energy $V_{bond,ij}$ [32]	[kcal/mol]	110.7	72.9	85.56
$r_{ij.0}$	[Å]	0.965	1.452	1.42
K_2_	-	532.5062	327.1657	400.3954
K_3_	-	−1282.905	−547.899	−835.1951
K_4_	-	2004.7658	526.5	1313.0142
Bond dissociation length $r_{ij}$	[Å]	1.4858	2.0717	1.9373

**Table 4:** Density of the molecular mixture at the end of the crosslinking simulation for *D_cut_* = 1.48 Å→2.5 Å at 150 °C, 170 °C, and 190 °C.

T [°C]	Density [g/cm^3^]					
	*D_cut_* = 1.48 Å	*D_cu_* = 1.5 Å	*D_cu_* = 1.6 Å	*D_cu_* = 1.7 Å	*D_cu_* = 1.8 Å	*D_cu_* = 2.5 Å
150	0.984	0.992	1.050	1.077	1.078	1.080
170	0.980	0.982	1.058	1.074	1.075	1.077
190	0.960	0.963	1.028	1.063	1.064	1.068

**Table 5:** Parameters in the reaction model estimated by regression with a confidence interval of 95 %.

**Method**	Expression of Reaction Model *f(α)*	$c_{\alpha }$	$n_{\alpha }$	$K_{\alpha }$	*logA* *[1/s]*	$R^{2}\;$	RMSE
*MD*	*nth*-order catalytic (*nOk*): $f\left(\alpha \right)=c_{\alpha }\cdot \;{\left(1-\alpha \right)}^{n_{\alpha }}\cdot \left(1+K_{\alpha }\cdot \alpha \right)$	0.3218	3.392	28.03	7.57	0.99	0.03
*DSC*		0.03	1.25	150.4	7.44	0.99	0.05
*DEA*		0.03	1.41	145.8	7.45	0.97	0.03

## Data Availability

The data presented in this study are available on request from the corresponding author.
